# Deciphering LAG-3: unveiling molecular mechanisms and clinical advancements

**DOI:** 10.1186/s40364-024-00671-0

**Published:** 2024-10-18

**Authors:** Alejandra Martínez-Pérez, Rocío Granda-Díaz, Candelaria Aguilar-García, Christian Sordo-Bahamonde, Segundo Gonzalez

**Affiliations:** 1 Department of Functional Biology, Immunology, Universidad de Oviedo, Oviedo, Spain; 2 Instituto Universitario de Oncología del Principado de Asturias (IUOPA), Oviedo, Spain; 3 Instituto de Investigación Sanitaria del Principado de Asturias (ISPA), Oviedo, Spain

**Keywords:** Immunotherapy, LAG-3, Checkpoint blockade, Relatlimab, Fianlimab, Favezelimab

## Abstract

Treatment based on immune checkpoint blockade has revolutionized cancer therapy. Despite the remarkable success achieved and the preclinical development of multiple checkpoint inhibitors targeting other checkpoints, only antibodies targeting the PD-1/PD-L1 axis and CTLA-4 have been approved for patient treatment, especially in solid tumors. Currently, with the approval of relatlimab, a LAG-3 blocking antibody, a third player, has been used in the fight against cancer. The endorsement of relatlimab marks a significant milestone in cancer immunotherapy, opening new avenues for combination therapies and enhancing treatment outcomes. However, the complex biology of LAG-3 may hinder its full development as a therapeutic alternative. In this review, we provide in-depth insight into the biology of LAG-3 and its current and future development in cancer treatment.

**Supplementary Information:**

The online version contains supplementary material available at 10.1186/s40364-024-00671-0.

## Introduction

Immunity is tightly regulated to avoid inappropriate or exacerbated responses, with immune checkpoints playing key roles in promoting or limiting immune responses. Immune checkpoint blockade (ICB)-based therapies hinge upon a delicate interplay of those costimulatory and coinhibitory signals orchestrated by immune checkpoints, which are crucial for maintaining immunological equilibrium. Although blocking antibodies targeting the PD-1/PD-L1 axis and CTLA-4 are considered cutting edge therapies in the fight against cancer because of their significant improvement in patient outcomes across a plethora of tumors, responses to such therapies often yield limited improvements. These findings underscore the need for additional targets and combination strategies to increase therapeutic efficacy.

The recent approval of relatlimab, a LAG-3 (lymphocyte-activation gene 3)-blocking antibody, marked a pivotal advancement in this field. Relatlimab introduces a new mechanism for modulating immune responses, broadening the arsenal of ICB therapies and opening new avenues for combination therapies to improve treatment outcomes.

However, the complexity of LAG-3 biology presents challenges that may hinder its full therapeutic exploitation. This review provides in-depth insight into the biology of LAG-3 and explores its current and future roles in cancer treatment. By elucidating the multifaceted functions of LAG-3, we aim to highlight its potential as a therapeutic target and continue efforts to integrate LAG-3 inhibitors into clinical practice.

## The biology of the LAG-3 immune checkpoint receptor

The checkpoint receptor LAG-3 (lymphocyte-activation gene 3) belongs to the immunoglobulin superfamily, and its expression pattern includes B lymphocytes, T lymphocytes, activated NK and NKT-like cells, Tregs and plasmacytoid dendritic cells (pDCs) [1–4]. This molecule is situated adjacent to CD4 on chromosome 12 owing to its very similar structure, with four extracellular domains (D1–D4), and shares an affinity for MHC class II molecules [5, 6]. Despite these similarities, their amino acid sequence is only 20% homologous, mainly in the regions encoding the extracellular region. The existing differences allow LAG-3 to bind to MHC class II molecules in different regions, and compared with CD4, which has a 100-fold affinity, LAG-3 also presents antagonistic functions [5, 7, 8]. This higher affinity depends on receptor dimerization through the D1 domain [9]. Notably, LAG-3 does not universally bind to MHC class II molecules but selectively binds to histocompatibility molecules that form a stable peptide presentation complex [10]. On the basis of these similarities, it was first hypothesized that LAG-3 and CD4 compete for binding to MHC class II molecules. Nevertheless, LAG-3 mutants that do not bind to HLA-II also resulted in diminished checkpoint responses; however, only cytoplasmic tail removal completely abrogated the LAG-3-mediated inhibitory response, suggesting that LAG-3 function relies mainly on its intracellular signaling, above its competition with CD4 for binding to class II HLAs [5, 11].

Since LAG-3 produces inhibitory signals in CD8 + T lymphocytes comparable to those mediated in CD4 + T lymphocytes, the expression of MHC class II molecules on antigen-presenting cells (APCs) could be a common inhibitory mechanism in both subpopulations, although cytotoxic T lymphocytes are not activated through antigen presentation by MHC class II molecules [10, 12]. However, given the restricted expression pattern of MHC class II compared with MHC class I under physiological conditions, the existence of other ligands for LAG-3 has been proposed. The first proposed alternative ligand was galectin-3, a lectin capable of modulating the T lymphocyte-mediated immune response through several mechanisms [13]. In vitro studies demonstrated that the LAG-3-galectin-3 interaction suppressed IFN-γ production by CD8 + T lymphocytes as well as the expansion of pDCs [14, 15]. Another potential LAG-3 ligand is LSECtin (liver and lymph node sinusoidal endothelial cell C-type lectin), whose expression in hepatic cells is inhibited upon LAG-3 binding, resulting in cytotoxic T-cell antiviral activity in murine models of hepatitis [16, 17]. In recent years, two new LAG-3 ligands have been proposed: FGL1 (fibrinogen-like protein 1) and α-synuclein. FGL1 is a soluble protein released by the liver and is capable of inhibiting T lymphocytes through LAG-3 but is independent of MHC class II [18]. Finally, the interaction between the pathological form of α-synuclein and LAG-3 in murine models of Parkinson’s disease appears to be an important mechanism in disease development; accordingly, treatment with a blocking monoclonal antibody could delay disease progression [19].

## Role of LAG-3 in immune response homeostasis

LAG-3 expression significantly increases as a consequence of immune response activation, and its rapid upregulation partly occurs because the molecule is stored in endosomes [20]. Its expression is induced as a result of TCR activation, as well as by cytokine stimulation (mainly by IL-12) [21, 22]. Upon activation, LAG-3 associates with the TCR:CD3 complex on the membrane of both CD4 + and CD8 + T lymphocytes and negatively regulates signal transduction induced by TCR activation, inhibiting proliferation and cytokine production [23]. Early studies suggested that LAG-3 leads to negative regulation of T lymphocyte-mediated responses. For example, LAG-3 blockade on CD4 + T lymphocytes resulted in increased production of IL-2, IL-4, IFN-γ, and TNFα, reinforcing the inhibitory role of this checkpoint [10, 24]. Similarly, LAG-3 knockout mice presented twice as many T lymphocytes as *wild-type mice did*, suggesting a role for this checkpoint in maintaining homeostasis [25]. LAG-3 expression in Treg lymphocytes has been reported to be essential for their suppressive functions [1]. These findings, along with the frequent coexpression of PD-1 and LAG-3, have associated the constitutive expression of this checkpoint with a state of exhaustion in both CD4 + and CD8 + T lymphocytes in patients with cancer and chronic infections [26–31]. Conversely, little is known about the role of LAG-3 in NK cells. Like T lymphocytes, increased expression of LAG-3 in NK cells is detected upon activation; nonetheless, no effect on NK cell-mediated cytotoxicity has been reported to date [22, 32]. Nevertheless, although LAG-3 blockade as monotherapy does not result in increased NK cell-mediated cytotoxicity, combination with IL-12 in murine models of breast cancer restored the antimetastatic activity of this immune subset [33]. It is noteworthy that a subpopulation of NKG2C + NK cells augments LAG-3 expression in response to IL-15 stimulation, which in turn is associated with enhanced PD-1 expression. Coexpression of both checkpoints, similar to T lymphocytes, might represent an exhausted NK cell phenotype [34]. Paradoxically, treatment with IMP321, a soluble form of the LAG-3 protein, led to increased activation and IFN-γ production by CD8 + T lymphocytes and NK cells in patients with advanced renal carcinoma, underscoring the need to explore the role of this checkpoint in NK cell-mediated responses [35, 36].

Currently, the exact mechanism mediated by LAG-3 signaling has not been elucidated. This checkpoint does not present the classic ITIM or ITAM domains found in other checkpoints, such as PD-1, CTLA-4, or BTLA. Instead, it possesses a cytoplasmic tail composed of three domains with no homology with other inhibitory receptors. The first motif consists of a serine phosphorylation region (two residues in humans and one in mice), but no function has yet been associated with it. The second motif, named “KIEELE” for the six amino acids it comprises, is known to be crucial for LAG-3-mediated inhibitory function, although the exact signaling mechanism triggered is currently unknown [11, 37]. Finally, the third motif consists of a repetition of glutamine and proline residues (EP) whose binding to LAG-3-associated protein (LAP) allows the checkpoint to colocalize with CD3, CD4, and/or CD8 molecules [38]. However, introducing mutations in this region did not affect LAG-3-mediated activity, suggesting that it may not be essential in the signaling pathway [11]. Three independent studies in murine models demonstrated the importance of the “KIEELE” motif in LAG-3-mediated signaling, both in vivo and in vitro [11, 37, 39]. However, there is controversy regarding the role of this region. In a recent study, deletion of the KIEELE region did not prevent LAG-3 inhibitory signaling, and the authors propose a new functional region named FxxL (which includes part of the KIEELE motif and the EP region) that could be crucial for these functions [40].

**Fig. 1:**
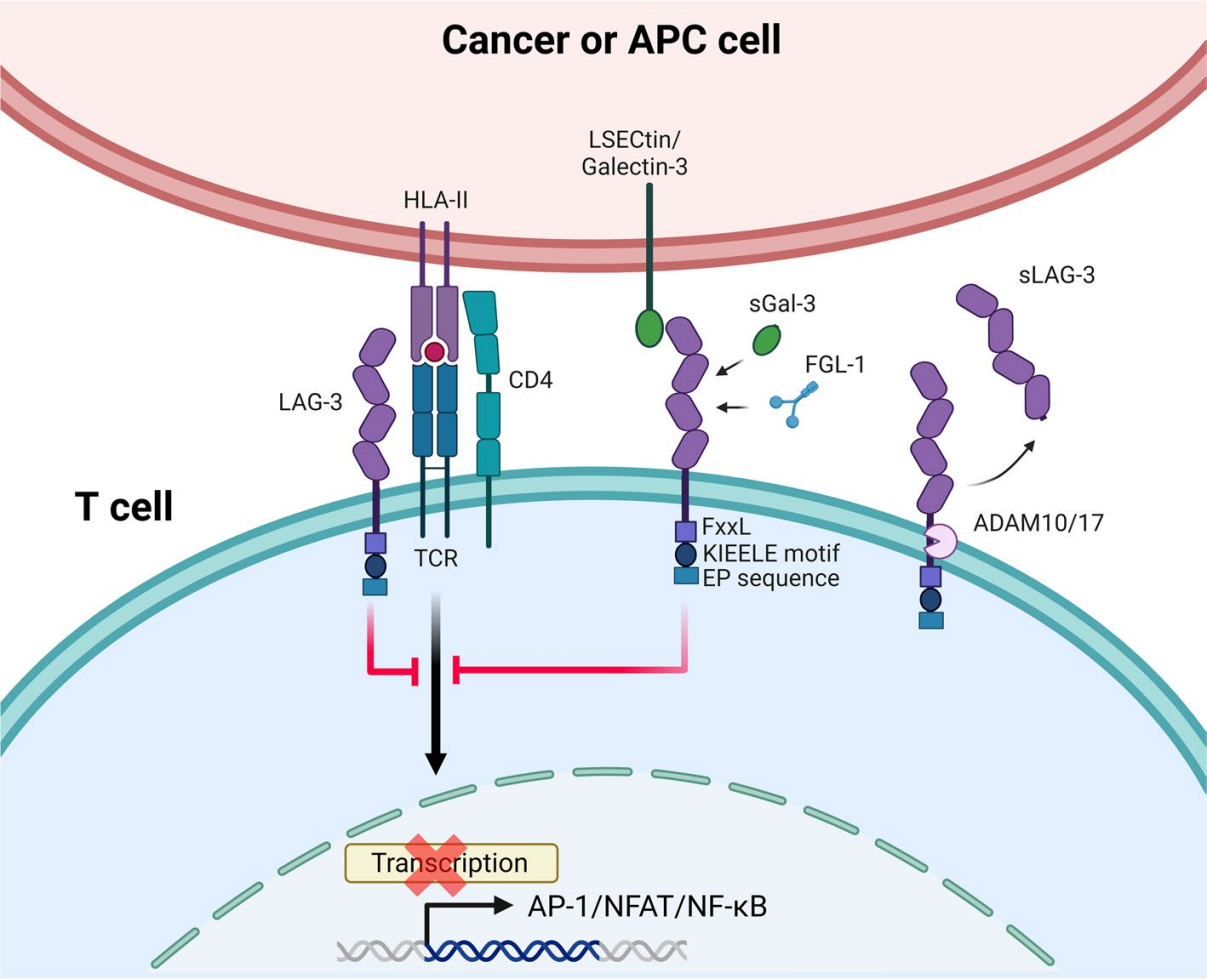
Mechanisms of LAG-3-mediated immune regulation in T cells. LAG-3 binds to MHC class II molecules on cancer cells or APCs, inhibiting TCR signaling and thus reducing the transcription of key transcription factors (AP-1, NFAT, and NF-κB). LAG-3 may also bind to other ligands, such as LSECtin/Galectin-3 and FGL-1, and its extracellular domain may be cleaved by ADAM10/17, producing soluble LAG-3 (sLAG-3). APC: antigen-presenting cell; MHC-II: MHC class II; sGAL-3: soluble galectin-3; FGL1: fibrinogen-like protein 1; LSECtin: liver sinusoidal endothelial cell lectin; LAG-3: lymphocyte-activation gene 3; TCR: T-cell receptor; sLAG-3: soluble LAG-3

The activity of LAG-3 is also regulated by cleavage of its extracellular region or shedding mediated by the metalloproteases ADAM10 and ADAM17, leading to the formation of its soluble form (sLAG-3) (Fig. 1) [9, 41]. Following T lymphocyte activation, there is a significant boost in the transcript levels of ADAM10 and ADAM17. Unlike ADAM10, which is constitutively transcribed and allows LAG-3 cleavage even in resting T lymphocytes, the removal of membrane LAG-3 by ADAM17 occurs only upon activation, and its activity is TCR- and PKC-dependent [9, 41].

Although no biological function has been described for sLAG-3 thus far, its cleavage is necessary for optimal T-cell function. Preventing the shedding of LAG-3 by generating mutants results in decreased T-cell proliferation and attenuated IL-2 and IFN-γ production due to checkpoint inhibitory signaling [41]. Similarly, the reduction in ADAM10 expression caused by the use of a retroviral shRNA vector significantly inhibited cell proliferation and IFN-γ release in a LAG-3-dependent manner [9, 41]. Taken together, these findings suggest that the removal of membrane LAG3 by ADAM10 and ADAM17 metalloproteases occurs via a negative feedback loop that allows the regulation of LAG-3 activity. Within the last few years, several biological activities have been described for sLAG-3, including immunostimulatory functions, leading to increased cytokine production (IL-12 and IFN-γ), dendritic cell maturation and T-cell proliferation [42–44]. Moreover, the soluble form has also been proposed as a biomarker for type I diabetes and even as a prognostic marker in tuberculosis and cancer [45, 46]. Nevertheless, the use of sLAG-3 as a prognostic factor is controversial, as elevated levels in patients’ serum have been associated with better prognosis in patients with gastric and breast cancers and with advanced disease, worse treatment-free survival and overall survival in patients with head and neck squamous cell carcinoma (HNSCC), hepatocellular carcinoma and chronic lymphocytic leukemia (CLL) [44, 47–51]. Moreover, higher levels of sLAG-3 in the serum of patients with advanced solid tumors before ICB-based therapies have been associated with poorer progression-free survival (PFS) and overall survival (OS) [43, 52].

## LAG-3 as a therapeutic target in *cancer*

In cancer, T lymphocytes are constantly exposed to antigen presentation, and an immunosuppressive microenvironment becomes exhausted, leading to a progressive loss of their antitumor capabilities associated with decreased cytokine production, proliferation, and the capacity of cytotoxic lymphocytes to eliminate tumor cells. The constitutive expression of LAG-3 in immune cells is associated with this state of exhaustion and has been linked to a weakened antitumor immune response in a wide variety of tumors, such as melanoma, Hodgkin’s lymphoma, CLL, and colorectal cancer [49, 53–55].

In the tumor microenvironment (TME) of several types of cancers, constitutive expression of LAG-3 has been observed in tumor-infiltrating lymphocytes (TILs), especially in CD4 + and CD8 + T lymphocytes, Treg cells, NK cells, NKT cells, and B lymphocytes, as well as in tumor-associated macrophages (TAMs) [2, 56–60]. The role of LAG-3 in immune evasion has been demonstrated in vitro and in vivo in multiple tumors. The use of a pharmacological inhibitor targeting glycogen synthase kinase-3α/β (GSK-3α/β) leads to decreased expression of LAG-3, which is associated with an increase in the level of the transcription factor T-bet, which binds to the LAG-3 promoter and prevents its transcription. Treatment with the inhibitor in combination with a LAG-3 blocking antibody produced a cooperative effect, resulting in increased tumor growth suppression, as well as increased levels of granzyme B and IFN-γ in CD8 + T lymphocytes in murine melanoma models [61]. Similarly, LAG-3 blockade delayed tumor development in HNSCC, NSCLC, and fibrosarcoma models [62, 63].

**Fig. 2:**
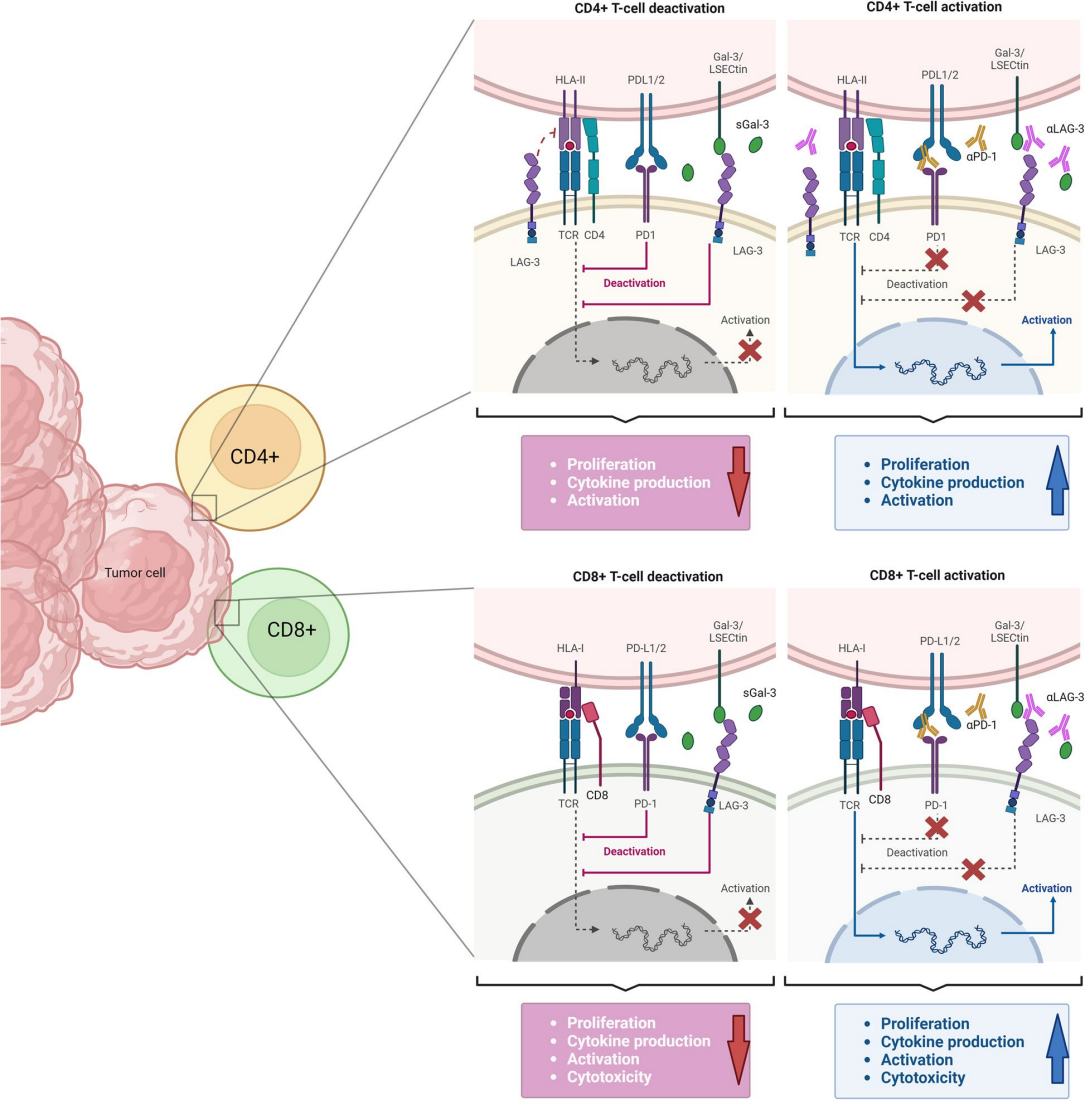
Current strategies targeting LAG-3 signaling in cancer

As mentioned above, FGL1 is released by hepatocytes in a soluble form under physiological conditions. However, its production has also been reported in tumor cells and is associated with poor prognosis and treatment resistance in different cancers, including hepatocellular carcinoma, gastric cancer, and lung cancer [18, 64–67]. Disruption of FGL1 and LAG-3 binding resulted in an enhanced antitumor immune response mediated by T lymphocytes, leading to tumor reduction in murine melanoma models [18]. Additionally, LSECtin expression in melanoma tumor cells and dendritic cells decreased the CD4 + and CD8 + T lymphocyte-mediated antitumor immune response through its interaction with LAG-3, clearly suggesting that LAG-3 inhibition may be triggered by multiple ligands that may be taken into consideration when developing therapies targeting this molecule (Fig. 2) [68].

Furthermore, the expression of LAG-3 is frequently associated with PD-1 expression and resistance to immunotherapy, which has prompted numerous studies exploring the combination of targeting both checkpoints [69]. Junko Matsuzaki et al*.* reported that approximately 80% of infiltrating CD8 + LAG-3 + T lymphocytes in ovarian cancer expressed PD-1. Combined treatment with blocking antibodies targeting both checkpoints resulted in increased antitumor activity of antigen-specific T lymphocytes in vitro and in murine models [70]. Consistently, a combination of PD-1 and LAG-3 blockade has been shown to promote an antitumor immune response in vitro and in vivo in several tumors, including melanoma, renal carcinoma, and CLL [71–73].

**Fig. 3:**
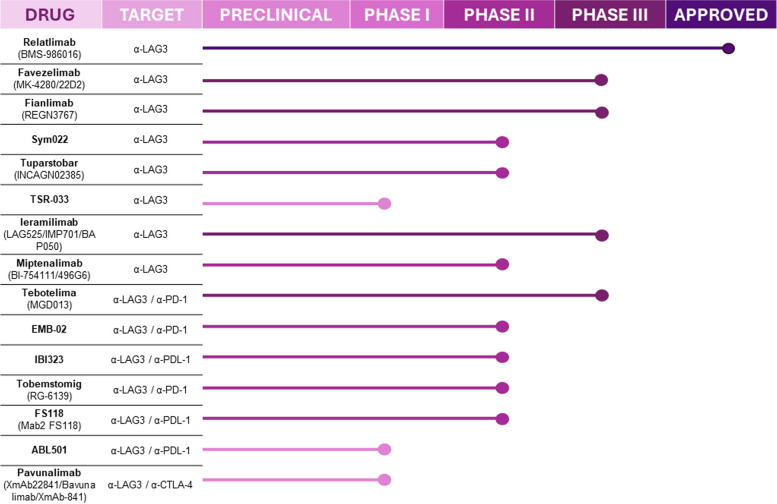
Current clinical development of LAG-3 targeting therapies in cancer

Despite the increasing number of ongoing clinical trials targeting LAG-3 in solid tumors (summarized in Fig. 3), scarce data are already available. Relatlimab (BMS-986016, Opdualag, Bristol-Myers Squibb Company), a first-in-class anti-LAG-3 blocking antibody, represents the third approval of ICB-based therapy for patients with cancer. In 2022, relatlimab in combination with nivolumab was approved by the Food and Drug Administration (FDA) and European Medicines Agency (EMA) for adult and pediatric patients with unresectable or metastatic melanoma. The results of the RELATIVITY-047 (NCT03470922) clinical trial with 714 patients demonstrated greater median progression-free survival in the relatlimab plus nivolumab cohort than in the nivolumab monotherapy cohort (10.1 *vs.* 4.6 months), as well as better progression-free survival (PFS) at 12 months (47.7% *vs.* 36%) [74]. The potential of LAG-3 blockade with relatlimab has also been explored in esophageal/gastroesophageal junction cancer and bladder and metastatic colorectal cancer [75–77]. Despite the encouraging results that led to the approval of relatlimab for the treatment of melanoma, results from the open-label phase II study RELATIVITY-060 showed a diminished overall response rate (ORR) in patients who underwent a combination regimen of relatlimab plus nivolumab and chemotherapy compared to nivolumab plus chemotherapy in patients with gastric cancer or gastroesophageal junction adenocarcinoma [78]. On the other hand, the phase II CheckMate 142 study presented promising results in patients with metastatic colorectal cancer treated with relatlimab and nivolumab, with an ORR of 50% and a PFS of 27.5 months, improving upon the response rates observed with anti-PD-1 monotherapy (ORR 31–33% and PFS of 16.5 months) [79–81].

In addition to relatlimab, fianlimab and favezelimab in combination with anti-PD-1 blocking antibodies are currently in phase III clinical trials testing their efficacy for the treatment of melanoma, NSCLC, and colorectal cancer [82–86]. More specifically, fianlimab in combination with cemiplimab (anti-PD-1 mAb) yielded a higher clinical response compared to monotherapy in patients with advanced melanoma (ORR of 61.2% and PFS 13.3 months *vs.* 11% and PFS 4.1 months), even in patients who received adjuvant anti-PD-1 therapy [87].

As a consequence of a better response to ICB combination regimens following LAG-3 blockade, several bispecific antibodies that target LAG-3/PD-1 or LAG-3/CTLA-4 blockade have been developed and are under clinical investigation in solid tumors. Among them, the bispecific antibody targeting PD-1 and LAG-3, tebotelimab, is the most advanced in clinical trials, with a phase III study in patients with HER2 + gastric cancer and other solid tumors. Specifically, approximately 30% of the patients exhibited a decreased tumor burden in the dose-escalation phase, whereas 34% (59 of 172) of the patients in the expansion cohort exhibited a decreased tumor burden [88]. On the other hand, a dose-escalation clinical trial in patients with hepatocellular carcinoma obtained limited results; only 1 out of 30 evaluable participants achieved a partial response and 14 had stable disease (median progression-free survival of 2.4–3.1 months) [89].

The use of bispecific antibodies is currently being studied as an alternative for patients who have become refractory to previous ICB-based therapies. It has been previously described (both at preclinical and clinical levels) that there is an increase in LAG-3 and PD-1 expression on TILs in anti–PD-1–resistant tumors, suggesting that targeting a single immune checkpoint might lead to the compensatory upregulation of alternative inhibitory receptors [90–93]. FS118, a tetravalent anti-LAG-3/anti-PD-L1 antibody, was evaluated in a dose-escalation and expansion clinical trial in patients with advanced/metastatic disease previously treated with anti-PD-1/PD-L1 mAbs. At the endpoint of the study, approximately 55% of the patients with acquired resistance to prior ICB-based therapy showed stable disease, reinforcing dual LAG-3/PD-(L)1 blockade as an alternative for re-challenging resistant tumors [94].

In Table 1, the current clinical development of LAG-3-targeting antibodies in solid tumors is summarized. A complete list of clinical trials targeting this immune checkpoint can be found in supplementary file.

**Table 1:**
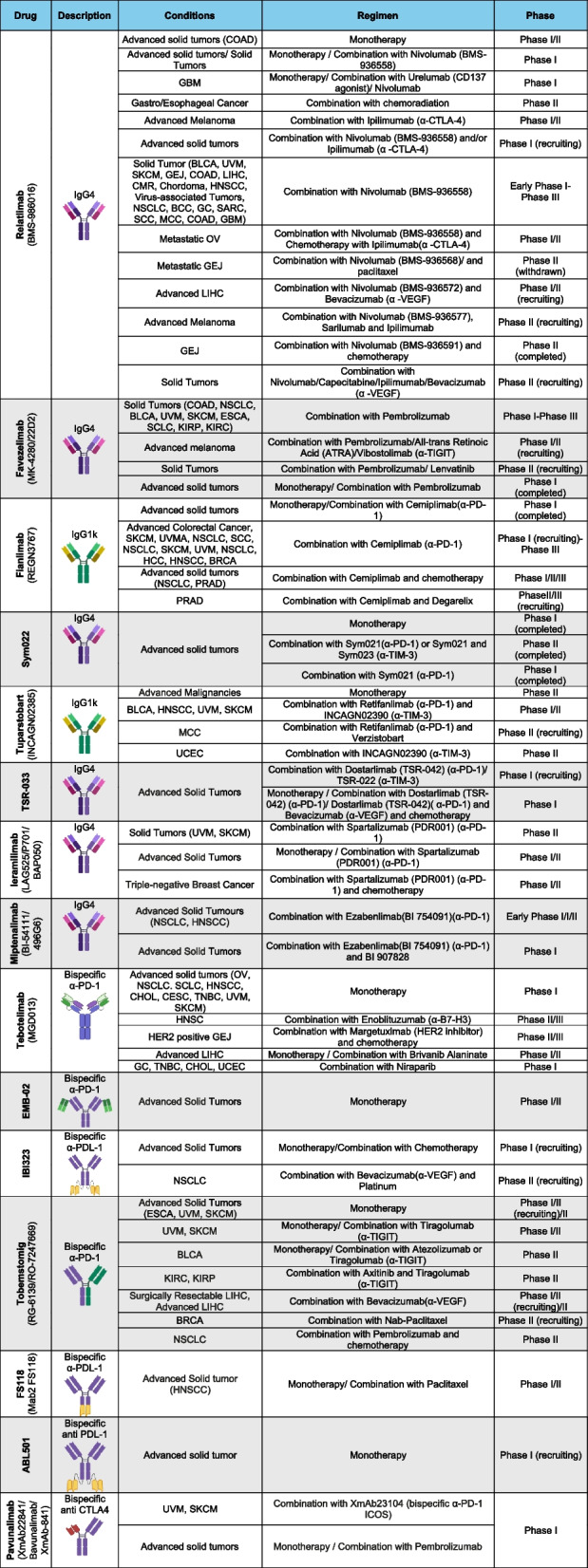
Clinical trials targeting LAG-3 in solid tumors

*BLCA* Bladder Urothelial Carcinoma, *BRCA* Breast invasive carcinoma, *CESC* Cervical squamous cell carcinoma and endocervical adenocarcinoma, *CHOL* Cholangiocarcinoma, *COAD* Colon adenocarcinoma, *ESCA* Esophageal carcinoma, *GC* Gastric Cancer, *GEJ* Gastroesophageal Junction Cancer, *HCC* hepatocellular carcinoma, *HNSCC* Head and Neck squamous cell carcinoma, *KIRC* Kidney renal clear cell carcinoma, *KIRP* Kidney renal papillary cell carcinoma, *LIHC* Liver hepatocellular carcinoma, *MCC* Merkel Cell Carcinoma, *NSCLC* Non-Small Cell Lung Cancer, *OV* Ovarian serous cystadenocarcinoma, *PRAD* Prostate adenocarcinoma, *SCC* Squamous Cell Carcinoma, *SCLC* Small Cell Lung Cancer, *SKCM* Skin Cutaneous Melanoma, *TNBC* Triple-negative Breast Cancer, *UCEC* Uterine Corpus Endometrial Carcinoma, *UVM* Uveal Melanoma

In the context of hematological cancers, LAG-3 expression has been correlated with the prognosis of CLL, acute myeloid leukemia (AML), follicular lymphoma (FL), and diffuse large B-cell lymphoma (DLBCL) patients [49, 58, 95–97]. In AML, the frequency of effector CD8 + T lymphocytes that coexpressed LAG-3 and PD-1 in bone marrow aspirates from patients was significantly greater than that in those from healthy donors [98]. Similar observations were made in patients with Hodgkin lymphoma and non-Hodgkin lymphoma, where CD4 + and CD8 + T lymphocytes in the peripheral blood expressed elevated levels of LAG-3. Deletion of CD4 + LAG-3 + T lymphocytes increased the antilymphoma responses of specific CD8 + T lymphocytes [54, 95]. In CLL, both LAG-3 expression in leukemic cells and its soluble form are increased, which is associated with a more aggressive clinical course [49, 50]. Remarkably, LAG-3 blockade, but not PD-1/PD-L1 axis blockade, enhances T-cell activation in CLL patients in vitro, in contrast with the findings of a study in which LAG3/PD-1 dual blockade increased the antileukemic immune response in Eµ-TCL1 murine models of CLL [49, 72].

These preclinical results have spurred numerous clinical trials currently exploring a LAG-3 blocking antibody in monotherapy but, notably, in combination with antibodies targeting the PD-1/PD-L1 axis. As mentioned above, relatlimab and other blocking mAbs are currently being tested in numerous phase I/II clinical trials for hematologic cancers, nonetheless, little data is available to date (Table 2). Favelizumab in combination with pembrolizumab was evaluated in patients with relapse/refractory Hodgkin lymphoma with prior anti-PD-1 therapy. The dual blockade led to an ORR of 31% and PFS rate at 12 months was 39%, showing clinical benefits in heavily pretreated patients [99].

**Table 2:**
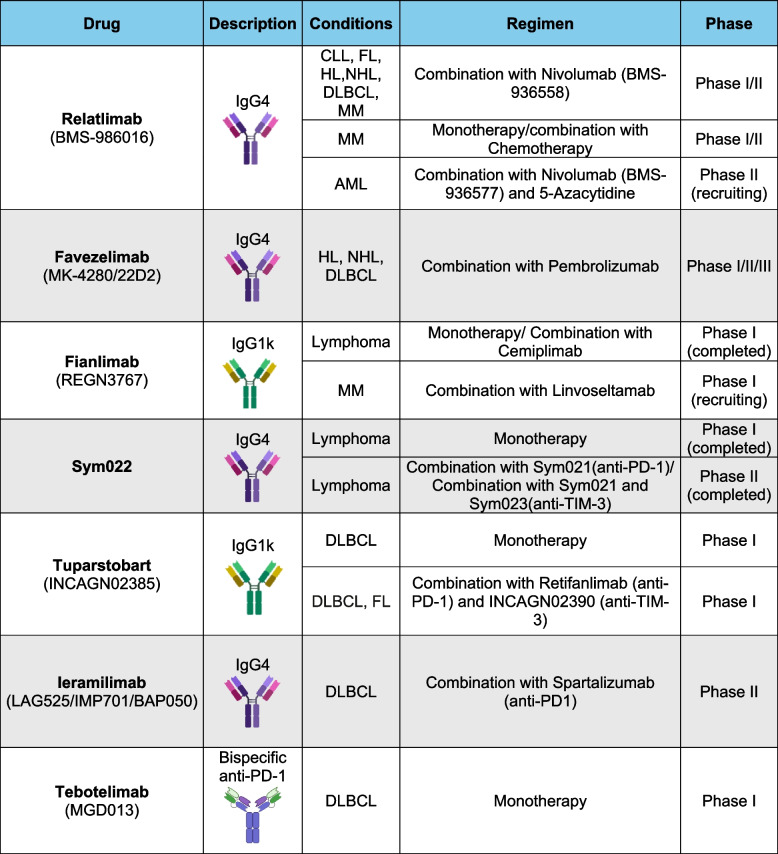
Clinical trials targeting LAG-3 in hematological cancers

In line with solid tumors, bispecific antibodies are being explored as potential tools in this type of cancers [100]. Notably, the bispecific antibody tebotelimab is also in clinical trials for hematological cancers, including DLBCL. Preliminary activity for tebotelimab was observed in 4 out of 11 patients with DLBCL (1 complete response and 3 partial responses), despite the lack of benefit from previous anti-PD-L1 and CAR-T-cell combination therapies. Upon treatment, patients showed an expansion of circulating CD3 + CD8 + T cell, as well as an increased on sera levels of IFN-γ, perforin and granzyme B, thus suggesting an enhanced immune-related antitumor response [88].

## Future perspectives and concluding remarks

LAG-3 plays a multifaceted role in immune response regulation and emerged as the third immune checkpoint established as a therapeutic target in cancer after the approval of PD-1/PD-L1 and CTLA-4. LAG-3 acts not only through direct interactions with MHC class II molecules but also by integrating into complex networks of inhibitory pathways, particularly in the tumor microenvironment. LAG-3’s co-expression with PD-1 on TILs has been shown to reinforce immune exhaustion, which is a hallmark of chronic viral infections and tumors. Importantly, the intracellular signaling cascade initiated by LAG-3 is unique compared to other checkpoints, lacking traditional ITIM or ITAM motifs, and instead relying on the ‘KIEELE’ motif for its inhibitory function. This non-canonical mechanism adds complexity to its regulatory role and suggests that LAG-3 modulates T-cell receptor (TCR) signaling in a manner distinct from PD-1.

A growing body of preclinical and clinical evidence underscores the potential of LAG-3 blockade as a viable strategy for enhancing antitumor immune responses, particularly when LAG-3 blockade is combined with existing immunotherapies targeting the PD-1/PD-L1 axis. Noteworthy, re-challenge with anti-PD-(L)1 mAbs along with LAG-3 blocking antibodies emerge as an interesting option to be explored.

Clinical trials investigating the efficacy of LAG-3 blocking antibodies, such as relatlimab, preferably in combination regimens, have demonstrated promising results across various hematological malignancies and solid tumors. Most combinatorial regimens currently being explored focus on the PD-1/PD-L1 axis, since it has been stablished as the gold standard of immunotherapy within the last few years. Nevertheless, despite the largely described coexpression of LAG-3 and PD-1 on immune cells, the presence of other immune checkpoints might also play an important role limiting antitumor responses. Several studies support that, along with PD-1, LAG-3 is coexpressed with TIM-3, TIGIT, IDO and VISTA in solid tumors and hematological malignancies [58, 101–107]. Notably, tiragolumab (TIGIT, phase I–III), Sym023 (TIM-3, phase I–III), HMBD-002 (VISTA, phase I) and BMS-986205 (IDO1, phase I–II), are also being explored as potential targets for cancer immunotherapy.

Notably, patients who undergo ICB-based therapies with anti-PD-(L)1 or anti-CTLA-4 frequently develop resistance. This acquired resistance may be associated with compensatory mechanisms that lead to the dysregulated expression of other inhibitory immune checkpoint receptors, including LAG-3. Preliminary clinical data suggest that re-challenging with anti-PD-(L)1 mAbs combined with LAG-3 blocking antibodies emerges as an intriguing option to be explored. Nevertheless, it remains unexplored whether other immune checkpoints are implicated in the aforementioned compensatory mechanisms, and the potential of novel and more complex combinatorial regimens could benefit patient outcomes.

In line with this, preclinical data of a novel LAG-3/TIGIT bispecific antibody have shown that reduced tumor growth is observed upon treatment, showing a synergistic effect when combined with nivolumab in murine models [108]. However, beyond PD-1/PD-L1, only a few clinical trials explore the potential combinations with anti-LAG-3 mAbs, such as TIM-3, and scarce data is available to date [109–111]. Therefore, despite clinical trials have focused on combinations with anti-PD-1/PD-L1 antibodies, preclinical data support the exploration of LAG-3 blockade along with novel immune checkpoint inhibitors alone or in combination with anti-PD-1/PD-L1. This approach could reveal new synergies for cancer immunotherapy development.

In accordance with this, chemoimmunotherapy has emerged as an option to explore. In addition to the classical view of the immunosuppressive role of chemotherapy, increasing preclinical and clinical evidence supports the immunostimulatory potential of these treatments under certain conditions. Notably, there has been an important increase in the number of FDA-approved chemotherapy and immunotherapy treatments for NSCLC, HNSCC, and breast and bladder cancer, among others [112–114]. Since LAG-3 blockade enhances anti-PD-1 therapy, several clinical trials are currently exploring the effect of combining chemotherapy with anti-PD-1 and anti-LAG-3 agents. This combination could offer a synergistic approach, enhancing the efficacy of each treatment modality and leading to improved patient outcomes. The integration of these therapies may maximize the antitumor response by simultaneously targeting multiple mechanisms of immune evasion and tumor cell survival.

In essence, understanding the fundamental role of LAG-3 in clinical development as a therapeutic target opens new avenues for improving patient outcomes. While mAbs such as relatlimab, fianlimab, and favezelimab have shown promise, their clinical efficacy remains heterogeneous across different tumor types. So, targeting LAG-3 still faces several challenges, and there are limitations to current LAG-3-targeted therapies that require further investigation, particularly regarding the differences in affinity and specificity between LAG-3 and its ligands (e.g., MHC-II *vs*. FGL-1). As mentioned earlier, FGL-1 is released by tumors to engage LAG-3 and dampen immune surveillance through non-MHCII-dependent mechanisms, which has been associated to resistance to current immunotherapies [115, 116]. Since most LAG-3 monoclonal antibodies under clinical development focus on disrupting the LAG-3-MHC-II axis, different cancers might exhibit varying responses to LAG-3 blockade. This highlights the importance of considering this complexity, particularly in tumors that highly express FGL-1, such as hepatocellular carcinoma and breast or gastric cancer. It remains necessary to evaluate whether limited responses observed in some clinical trials in hepatocellular carcinoma might be related to the importance of different LAG-3 ligands within distinct tumors [89].

Within the last few years, novel ways of targeting immune checkpoints arise beyond antibodies. Small molecule inhibitors (SMI) present a promising alternative to monoclonal antibodies for targeting immune checkpoints like LAG-3 due to several advantages. These include their potential for oral administration, improved tumor penetration due to their smaller size, and reduced production costs. Unlike antibodies, SMI offer more convenience and could be more widely accessible. Furthermore, they can be designed to simultaneously inhibit multiple immune checkpoint pathways, which may enhance therapeutic efficacy by addressing compensatory mechanisms that contribute to resistance [117, 118]. Despite the development of SMI has focused on PD-1/PD-L1 (with several phase I/II clinical trials currently ongoing), inhibitors targeting LAG-3 have already been described. This SMI dualistically inhibit LAG-3 binding to both MHC-II and FGL1, which could help achieve a more comprehensive blockade of LAG-3 signaling [61, 119]. Future research should focus on optimizing the specificity and bioavailability of these inhibitors, as well as assessing their efficacy in combination with both checkpoint inhibitors and traditional chemotherapies.

In conclusion, LAG-3 has been established as the third member of immune checkpoint blockade (ICB)-based therapies, supported by solid and promising *real-world* data. However, several challenges remain, including a better understanding of LAG-3 signaling pathways, the influence of different binding partners, and the expansion of combinatorial regimens within the immunotherapy portfolio.

## Supplementary Information

Supplementary Material 1:

## Data Availability

No datasets were generated or analysed during the current study.
