# Correction: Deciphering LAG-3: unveiling molecular mechanisms and clinical advancements

**DOI:** 10.1186/s40364-024-00683-w

**Published:** 2024-11-11

**Authors:** Alejandra Martínez-Pérez, Rocío Granda-Díaz, Candelaria Aguilar-García, Christian Sordo-Bahamonde, Segundo Gonzalez

**Affiliations:** 1 Department of Functional Biology, Immunology, Universidad de Oviedo, Oviedo, Spain; 2 Instituto Universitario de Oncología del Principado de Asturias (IUOPA), Oviedo, Spain; 3 Instituto de Investigación Sanitaria del Principado de Asturias (ISPA), Oviedo, Spain

**Correction: Biomarker Research (2024) 12:126**

10.1186/s40364-024-00671-0

The authors wish to revise the Funding statement to note the contribution of the European Union. The Funding statement should instead read as follows:

**Funding**

This work was supported by a Spanish grant from Instituto de Salud Carlos III (PI23/01576) co-funded by the European Union, Fundación Xti Xtod@s and Fundación Alimerka. R.G-D. holds an Intramural ISPA-Janssen grant, and A.M-P. holds an Intramural ISPA-FINBA grant. C.A.-G. holds a fellowship from Fundación Científica de la Asociación Española Contra el Cáncer (PRDAS222516AGUI), Spain.

