# Supplementary material for: Deciphering LAG-3: unveiling molecular mechanisms and clinical advancements
Source: Biomark Res. 2024 Oct 18;12:126. doi: 10.1186/s40364-024-00671-0 (PMC11487938; doi:10.1186/s40364-024-00671-0)
Supplement: Supplementary file 1 — Supplementary Material 1: [file 40364_2024_671_MOESM1_ESM.docx]

| **Drug** | **Conditions** | **Regimen** | **Phase** | **ID** |
| --- | --- | --- | --- | --- |
| **Relatlimab**  (BMS-986016) | Advanced solid tumors | Monotherapy | Phase II (recruiting) | NCT03607890 |
|  | Advanced solid tumors | Combination with Nivolumab (BMS-936558) | Phase I | NCT05498480 |
|  | Urothelial cancer | Combination with Nivolumab (BMS-936558) | Phase II (recruiting) | NCT06237920 |
|  | Solid tumors | Monotherapy / Combination with Nivolumab (BMS-936558) | Phase I/II | NCT01968109 |
|  | Metastatic Melanoma | Combination with Nivolumab (BMS-936558) | Phase II | NCT03743766 |
|  | Metastatic gastroesophageal cancer | Combination with Nivolumab (BMS-936558) | Phase I/II | NCT03610711 |
|  | Advanced Colorectal Cancer | Monotherapy | Phase II | NCT03642067 |
|  | Advanced solid tumors | Monotherapy / Combination with Nivolumab (BMS-936558) | Phase I | NCT02966548 |
|  | Colorectal cancer | Combination with Nivolumab (BMS-936558) | Phase II | NCT03867799 |
|  | Advanced tumors | Combination with Nivolumab (BMS-936558) | Phase I | NCT04112498 |
|  | Melanoma with brain metastases | Combination with Nivolumab (BMS-936558) | Phase II (recruiting) | NCT05704647 |
|  | Metastatic Uveal Melanoma | Combination with Nivolumab (BMS-936558) | Phase II | NCT04552223 |
|  | Advanced Liver Cancer | Combination with Nivolumab (BMS-936558) | Phase II | NCT04567615 |
|  | Metastatic Colorectal Cancer | Combination with Nivolumab (BMS-936558) | Phase III | NCT05328908 |
|  | Advanced Melanoma | Combination with Nivolumab (BMS-936558) | Phase II/III | NCT03470922 |
|  | Renal Medullary Carcinoma | Combination with Nivolumab (BMS-936558) | Phase II (recruiting) | NCT05347212 |
|  | Resectable Hepatocellular Carcinoma | Combination with Nivolumab (BMS-936558) | Phase I (recruiting) | NCT04658147 |
|  | Advanced Melanoma | Combination with Ipilimumab | Phase II | NCT03978611 |
|  | Untreated Metastatic or Unresectable Melanoma | Combination with Nivolumab (BMS-936558) | Phase III (recruiting) | NCT05625399 |
|  | Advanced solid tumors | Combination with Nivolumab (BMS-936558) and/or Ipilimumab | Phase I/II | NCT03459222 |
|  | Advanced Chordoma | Combination with Nivolumab (BMS-936558) | Phase II (completed) | NCT03623854 |
|  | Hodgkin and Non-Hodgkin Lymphoma | Combination with Nivolumab (BMS-936558) | Phase I/II | NCT05255601 |
|  | Metastatic Melanoma | Combination with Nivolumab (BMS-936558) | Phase II | NCT03724968 |
|  | Melanoma | Combination with Nivolumab (BMS-936558) | Phase II (recruiting) | NCT06101134 |
|  | Melanoma | Combination with Nivolumab (BMS-936558) | Phase II (recruiting) | NCT05912244 |
|  | Gastro/​Esophageal Cancer | Combination with chemoradiation | Phase I | NCT03044613 |
|  | Head and Neck Cancer | Combination with Nivolumab (BMS-936558) | Phase II (recruiting) | NCT04080804 |
|  | Virus-associated Tumors | Combination with Nivolumab (BMS-936558) | Phase I/II (completed) | NCT02488759 |
|  | Primary Clear Cell Renal Cell Cancer | Combination with Nivolumab (BMS-936558) | Phase II (recruiting) | NCT05148546 |
|  | Advanced Non-Small Cell Lung Cancer | Combination with Nivolumab (BMS-936558) | Phase II (terminated) | NCT02750514 |
|  | Advanced Renal Cell Carcinoma | Combination with Nivolumab (BMS-936558) | Phase II (completed) | NCT02996110 |
|  | Advanced Liver Cancer | Combination with Nivolumab (BMS-936572) and Bevacizumab | Phase I/II (recruiting) | NCT05337137 |
|  | Metastatic Gastric Cancer or Gastroesophageal Junction Adenocarcinoma | Combination with Nivolumab (BMS-936568)/ and paclitaxel | Phase II (withdrawn) | NCT03704077 |
|  | Solid tumors | Combination with Nivolumab (BMS-936569) and Ipilimumab | Phase I (recruiting) | NCT05176483 |
|  | Melanoma | Combination with Nivolumab (BMS-936558) | Phase III | NCT06246916 |
|  | Metastatic Basal Cell Carcinoma | Combination with Nivolumab (BMS-936558) | Phase II (recruiting) | NCT03521830 |
|  | Locoregionally Advanced Melanoma | Combination with Nivolumab (BMS-936558) | Early Phase I (recruiting) | NCT06295159 |
|  | Advanced Gastric Cancer | Combination with Nivolumab (BMS-936558) | Phase II (completed) | NCT02935634 |
|  | Metastatic Soft-tissue Sarcoma | Combination with Nivolumab (BMS-936558) | Phase II (recruiting) | NCT04095208 |
|  | Squamous Cell Carcinoma of the Skin | Combination with Nivolumab (BMS-936558) | Phase II (recruiting) | NCT04204837 |
|  | Head and neck  squamous cell carcinoma | Combination with Nivolumab (BMS-936558) | Phase II | NCT04326257 |
|  | Advanced Melanoma | Combination with Nivolumab (BMS-936577), Sarilumab and Ipilimumab | Phase II (recruiting) | NCT05428007 |
|  | Metastatic Uveal Melanoma | Combination with Nivolumab (BMS-936558) | Phase I | NCT04935229 |
|  | AML | Combination with Nivolumab (BMS-936577) and 5-Azacytidine | Phase II (recruiting) | NCT04913922 |
|  | Metastatic Urothelial Cancer | Combination with Nivolumab (BMS-936558) | Phase II (recruiting) | NCT04953104 |
|  | Advanced Melanoma | Combination with Nivolumab (BMS-936558) | Phase III | NCT05002569 |
|  | Advanced Melanoma | Combination with Nivolumab (BMS-936558) | Phase III | NCT06112314 |
|  | Refractory B-Cell Malignancies | Combination with Nivolumab (BMS-936558) | Phase I/II (completed) | NCT02061761 |
|  | Metastatic Melanoma | Combination with Nivolumab (BMS-936558) | Phase II (recruiting) | NCT05034536 |
|  | Melanoma | Combination with Nivolumab (BMS-936558) | Phase III (recruiting) | NCT05418972 |
|  | Refractory B-cell Malignancies | Combination with Nivolumab (BMS-936558) | Phase I/II (completed) | NCT03310619 |
|  | Melanoma with brain metastases | Combination with Nivolumab (BMS-936558) | Early Phase I | NCT05704933 |
|  | Advanced Non-Small Cell Lung Cancer | Combination with Nivolumab (BMS-936558) | Phase II | NCT04623775 |
|  | Metastatic Ovarian Cancer | Combination with Nivolumab (BMS-936558) and Chemotherapy with or without Ipilimumab | Phase I/II | NCT04611126 |
|  | Solid tumors | Combination with Nivolumab (BMS-936558) | Phase I (completed) | NCT03335540 |
|  | Multiple mieloma | Monotherapy or combination and chemotherapy | Phase I/II | NCT04150965 |
|  | Merkel Cell Carcinoma | Combination with Nivolumab (BMS-936558) | Phase II (recruiting) | NCT06151236 |
|  | Early-stage Colon Cancer | Combination with Nivolumab (BMS-936558) | Phase II (recruiting) | NCT03026140 |
|  | Advanced Melanoma | Combination with Nivolumab (BMS-936558) | Phase III | NCT06264180 |
|  | Cutaneous Squamous Cell Carcinoma | Combination with Nivolumab (BMS-936558) | Phase II | NCT06288191 |
|  | Melanoma | Combination with Nivolumab (BMS-936558) | Phase II (completed) | NCT02519322 |
|  | Advanced gastric cancer | Combination with Nivolumab (BMS-936558) | Phase II | NCT04062656 |
|  | Advanced Melanoma | Combination with Nivolumab (BMS-936558) | Phase I (recruiting) | NCT04697576 |
|  | Resectable Non-small Cell Lung Cancer | Combination with Nivolumab (BMS-936558) | Phase II (recruiting) | NCT04205552 |
|  | Gastric or Gastroesophageal Junction (GEJ) Cancers | Combination with Nivolumab (BMS-936591) and chemotherapy | Phase II (completed) | NCT03662659 |
|  | Recurrent glioblastoma | Combination with Nivolumab (BMS-936558) | Phase II | NCT06325683 |
|  | Metastatic Uveal Melanoma | Combination with Nivolumab (BMS-936558) | Phase II (recruiting) | NCT05077280 |
|  | Colorectal cancer | Combination with Nivolumab (BMS-936558) | Phase II | NCT02060188 |
|  | Refractary solid tumors, lymphoma, Multiple Myeloma | Combination with Nivolumab (BMS-936558) | Phase II | NCT02465060 |
|  | Glioblastoma | Monotherapy/Urelumab/Nivolumab | Phase I | NCT02658981 |
|  | Glioblastoma | Combination with Nivolumab (BMS-936558) | Phase I | NCT03493932 |
|  | Pan-tumor | Combination with Nivolumab/Capecitabine/Ipilimumab/Bevacizumab | Phase II (recruiting) | NCT03899155 |
|  | Urothelial Carcinoma | Combination with Nivolumab (BMS-936558) | Phase II/III (recruiting) | NCT05987241 |
|  | Nasopharyngeal Carcinoma | Combination with Nivolumab (BMS-936558) | Phase II (recruiting) | NCT06029270 |
|  | advanced or refractory Lymphoma, multiple myeloma, solid tumors | Combination with Nivolumab (BMS-936558) | Phase II | NCT06400264 |
| **Favezelimab**  (MK-4280/22D2) | Hematologic Malignancies or Solid Tumors | Combination with Pembrolizumab | Phase I/II | NCT06395090 |
|  | Hematologic Malignancies | Combination with Pembrolizumab | Phase I/II (recruiting) | NCT03598608 |
|  | Advanced Solid Tumors | Monotherapy/ Combination with Pembrolizumab | Phase I (completed) | NCT02720068 |
|  | Solid tumors | Combination with Pembrolizumab/lenvatinib | Phase II (recruiting) | NCT06036836 |
|  | Metastatic Colorectal cancer | Combination with Pembrolizumab | Phase III | NCT05600309 |
|  | Metastatic Colorectal cancer | Combination with Pembrolizumab | Phase III | NCT05064059 |
|  | Advanced Non-Small Cell Lung Cancer | Combination with Pembrolizumab | Phase II | NCT03516981 |
|  | Hodgkin Lymphoma | Combination with Pembrolizumab | Phase III (recruiting) | NCT05508867 |
|  | Metastatic Urothelial Cancer | Combination with Pembrolizumab | Phase I/II | NCT05845814 |
|  | Invasive bladder cancer | Combination with Pembrolizumab | Phase II (recruiting) | NCT02625961 |
|  | Advanced Colorectal Cancer | Combination with Pembrolizumab | Phase II | NCT04895722 |
|  | Advanced melanoma | Combination with Pembrolizumab | Phase I/II | NCT04303169 |
|  | Advanced melanoma | Combination with Pembrolizumab/All-trans Retinoic Acid (ATRA)/Vibostolimab (anti-TIGIT) | Phase I/II (recruiting) | NCT04305054 |
|  | Advanced esophageal cancer | Combination with Pembrolizumab | Phase I/II | NCT05342636 |
|  | Advanced Small Cell Lung Cancer | Combination with Pembrolizumab | Phase I/II | NCT04938817 |
|  | Renal Cell Carcinoma | Combination with Pembrolizumab | Phase I/II | NCT04626518 |
|  | Renal Cell Carcinoma | Combination with Pembrolizumab | Phase I/II | NCT04626479 |
| **Fianlimab**  (REGN3767) | Advanced cancers | Monotherapy/ Combination with Cemiplimab (antiPD-1) | Phase I (completed) | NCT03005782 |
|  | Advanced Colorectal Cancer | Combination with Cemiplimab | Phase II (recruiting) | NCT06205836 |
|  | Advanced Melanoma | Combination with Cemiplimab | Phase II/III | NCT06190951 |
|  | Advanced Non-Small Cell Lung Cancer | Combination with Cemiplimab | Phase II/III (recruiting) | NCT05785767 |
|  | Advanced Cutaneous Squamous Cell Carcinoma | Combination with Cemiplimab | Phase II | NCT06384820 |
|  | Lung Cancer | Combination with Cemiplimab | Phase I (recruiting) | NCT03233139 |
|  | Melanoma | Combination with Cemiplimab | Phase III (recruiting) | NCT05608291 |
|  | Metastatic Melanoma | Combination with Cemiplimab | Phase III | NCT06246916 |
|  | Advanced Melanoma | Combination with Cemiplimab | Phase III | NCT05352672 |
|  | Resectable NSCLC, HCC, and HNSCC | Combination with Cemiplimab | Phase II (recruiting) | NCT03916627 |
|  | Advanced Non-small Cell Lung Cancer | Combination with Cemiplimab and chemotherapy | Phase II/III (recruiting) | NCT05800015 |
|  | Prostate Cancer | Combination with Cemiplimab and chemotherapy | Phase II (recruiting) | NCT06161441 |
|  | Prostate Cancer | Combination with Cemiplimab and Degarelix | Phase II/III (recruiting) | NCT04989946 |
|  | Multiple mieloma | Combination with Linvoseltamab | Phase I (recruiting) | NCT05137054 |
|  | Advanced solid tumors | Combination with Cemiplimab and chemotherapy | Phase I/II | NCT04706715 |
|  | Breast cancer | Combination with Cemiplimab | Phase II | NCT01042379 |
| **Sym022** | Tumor malignancies or Lymphomas | Monotherapy | Phase I (completed) | NCT03489369 |
|  |  | Combination with Sym021/ Combination with Sym021 and Sym023 | Phase II (completed) | NCT03311412 |
|  | Advanced solid tumors | Combination with Sym021/ Combination with Sym021 | Phase I (completed) | NCT04641871 |
| **Tuparstobart** (INCAGN02385) | Merkel Cell Carcinoma | Combination with retifanlimab and verzistobart | Phase 2 Recruiting | NCT06056895 |
|  | Advanced Malignancies | Monotherapy | Phase I | NCT03538028 |
|  | Melanoma | Combination with INCAGN02390 and INCMGA00012 | Phase I/II | NCT04370704 |
|  | DLBCL, FL | Combination with Retifanlimab and INCAGN02390 | Phase1 | NCT06290622 |
|  | Head and Neck Cancer | Combination with Retifanlimab and INCAGN02390 | Phase II | NCT05287113 |
|  | Urothelial Carcinoma | Combination with Retifanlimab and INCAGN02390 | Phase II | NCT04586244 |
|  | Endometrial Cancer | Combination with INCAGN02390 | Phase II | NCT04463771 |
| **TSR-033** | Advanced Solid Tumors | Monotherapy/ Combination with Dostarlimab (TSR-042)/ Dostarlimab/ Bevacizumab /chemotherapy | Phase I | NCT03250832 |
|  |  | Combination with Dostarlimab (TSR-042)and Plus TSR-022 | Phase I (recruiting) | NCT02817633 |
| **Ieramilimab** (LAG525/IMP701/BAP050) | Melanoma | Combination with Spartalizumab (PDR001) | Phase II | NCT03484923 |
|  | Advanced Solid Tumors | Monotherapy / Combination with Spartalizumab (PDR001) | Phase I/II | NCT02460224 |
|  | Advanced Triple-negative Breast Cancer | Combination with Spartalizumab (PDR001)/ Carboplatin | Phase II | NCT03499899 |
|  | Solid and Hematologic Malignancies | Combination with Spartalizumab (PDR001) | Phase II | NCT03365791 |
|  | Triple-negative Breast Cancer | Combination with Spartalizumab (PDR001) and chemotherapy | Phase I/II | NCT03742349 |
| **Miptenalimab**  (BI-754111/496G6) | Advanced Non-small Cell Lung Cancer and Head and Neck Squamous Cell Carcinoma | Combination with Ezabenlimab (BI 754091) | Phase I | NCT03780725 |
|  | Advanced Solid Tumours | Combination with Ezabenlimab (BI 754091) | Early Phase I | NCT03433898 |
|  | Advanced Solid Tumors | Combination with Ezabenlimab (BI 754091) and BI 907828 | Phase I | NCT03964233 |
|  | Non-small Cell Lung Cancer and Other Solid Tumors | Combination with Ezabenlimab (BI 754091) | Phase I | NCT03156114 |
|  | Advanced Solid Tumors | Combination with Ezabenlimab (BI 754091) | Phase II | NCT03697304 |
| **Tebotelimab**  (MGD013) | Head and Neck Cancer | Combination with Enoblituzumab | Phase II | NCT04634825 |
|  | HER2-Positive Gastric or Gastroesophageal Junction Cancer | Combination with Margetuximab and chemotherapy | Phase II/III | NCT04082364 |
|  | Unresectable or Metastatic Neoplasms | Monotherapy | Phase I | NCT03219268 |
|  | Advanced Liver Cancer | Monotherapy/ Combination with Brivanib Alaninate | Phase I/II | NCT04212221 |
|  | Melanoma | Monotherapy | Phase I | NCT04653038 |
|  | Head and Neck Cancer | Combination with enoblituzumab | Phase II/III | NCT04129320 |
|  | Solid Tumor | Combination with Niraparib | Phase I | NCT04178460 |
| **EMB-02** | Advanced Solid Tumors | Monotherapy | Phase I/II | NCT04618393 |
| **IBI323** | Advanced Malignancies | Monotherapy/ Combination with Chemotherapy | Phase I (recruiting) | NCT04916119 |
|  | Non-Small Cell Lung Cancer | Combination with Bevacizumab/Platinum | Phase II (recruiting) | NCT05296278 |
| **Tobemstomig**  (RG-6139/RO-7247669) | Urothelial Cancer | Monotherapy/ Combination with Atezolizumab/Tiragolumab | Phase II | NCT05645692 |
|  | Advanced Liver Cancers | Monotherapy/Atezolizumab/Tiragolumab/Bevacizumab/Tocilizumab/TPST-1120/ADG126/io-108/NKT2152 | Phase I/II (recruiting) | NCT04524871 |
|  | Breast cancer | Combination with Nab-Paclitaxel | Phase II (recruiting) | NCT05852691 |
|  | Non-Small Cell Lung Cancer | Combination with Pembrolizumab/Paclitaxel/Pemetrexed/Carboplatin | Phase II | NCT05775289 |
|  | Advanced or Metastatic Esophageal Squamous Cell Carcinoma | Monotherapy | Phase II | NCT04785820 |
|  | Renal Cell Carcinoma | Combination with Axitinib/Tiragolumab | Phase II | NCT05805501 |
|  | Surgically Resectable Hepatocellular Carcinoma | Combination with Bevacizumab | Phase I/II (recruiting) | NCT05908786 |
|  | Advanced and/or Metastatic Solid Tumors | Monotherapy | Phase I/II (recruiting) | NCT04140500 |
|  | Unresectable or Metastatic Melanoma | Monotherapy | Phase I/II (recruiting) | NCT05419388 |
|  | Melanoma | Monotherapy/ Combination with Tiragolumab | Phase I/II | NCT05116202 |
| **FS118** (Mab2 FS118) | Squamous Cell Carcinoma of Head and Neck/Advanced Malignancies | Monotherapy/ Combination with Paclitaxel | Phase I/II | NCT03440437 |
| **ABL501** | Advanced Solid Tumor | Monotherapy | Phase I (recruiting) | NCT05101109 |
| **Pavunalimab** (XmAb22841/ Bavunalimab/ XmAb-841) | Melanoma | Combination with XmAb23104 (bispecific PD1 ICOS) | Phase I | NCT05695898 |
|  | advanced solid tumors | Monotherapy/ Combination with Pembrolizumab | Phase I | NCT03849469 |
